# Scoping Review of Co-Design in Mental Health Research: Essential Elements and Recommendations

**DOI:** 10.1177/15394492251367259

**Published:** 2025-09-08

**Authors:** Helena Roennfeldt, Victoria Stewart, Marianne Wyder, Teresa Fawcett, Caroline Robertson, Rebecca Soole, Dan Siskind, Amanda Wheeler, Justin Chapman

**Affiliations:** 1 Griffith University, Brisbane, Queensland, Australia; 2 Griffith University, Gold Coast, Queensland, Australia; 3 Metro South Addiction and Mental Health Service, Brisbane, Queensland, Australia; 4 The University of Queensland, Brisbane, Australia; 5 Queensland Centre for Mental Health Research, Brisbane, Australia; 6 University of Auckland, New Zealand

**Keywords:** scoping reviews, mental health, communities

## Abstract

Co-design is increasingly being adopted within mental health service design and delivery, but is less common in research. Co-design ensures that research is relevant and benefits people accessing services. This review examined frameworks, models, and/or guidelines for co-designing mental health research, asking: (a) How is co-design defined? (b) What are the elements, values, and design tools? (c) What recommendations are proposed? A scoping review of peer-reviewed and gray literature on co-design in mental health research was undertaken and narratively synthesized. A total of 28 studies were included, showing varied understandings of co-design. Key values included social justice, recognizing lived experience as expertise, and fostering safe and trusting relationships. Traditional academic structures often hinder co-design; however, innovative research methods have shown potential. Recommendations and strategies to overcome barriers are provided. To enhance the adoption of co-design in mental health research, clearer terminology and agreed-upon values and processes are needed.

## Plain Language Summary

Enhancing Co-Design in Mental Health Research: A Review of Best Practices and Guidelines

Co-design in mental health research means working together with people who have personal experiences of mental health diagnoses and service use to create better solutions. Co-design is often used to improve mental health services, but it is not used as much in research. This study looked at articles and reports to understand how co-design is explained in mental health research, what key ideas and values it includes, and how to do co-design well. The authors found 28 studies that addressed co-design. They noticed that different words are used for co-design and that people have different ideas about how to use it in research. They found that to make co-design work well, it is important to be fair, listen to people who have personal experience, and build strong, trusting relationships. Sometimes, the way research is usually done can make co-design harder, but there are creative ways to make it better. The authors found that clearer and more consistent language and steps are needed so co-design can be used more effectively in mental health research. They also share ideas on how to fix problems that get in the way of co-design. This helps make research more useful and helpful for people using mental health services.

## Introduction

Co-design belongs to a collection of approaches broadly described as participatory methods aimed at increasing the involvement and leadership of people with lived experience (Grindell et al., 2022; Palmer et al., 2019). These approaches are known by various terms globally, such as participatory action research, co-design, co-production and patient and public involvement (Vargas et al., 2022). Co-design has its roots in participatory design, arising in Scandinavia in the 1970s and broadly refers to a participatory approach to designing solutions (Sanders & Stappers, 2008). It builds on social democracy and community development principles, encouraging all critical stakeholders to participate as respected equal partners (NSW Council of Social Service, 2017). A key tenet of co-design is centering those affected by the problem in the design process and recognizing them as *experts by experience* (McKercher, 2021). Co-design has gained attention in health settings due to policy imperatives for greater consumer involvement and recognition of the value of lived experience knowledge in informing service delivery and reform (Slattery et al., 2020). However, a lack of consensus remains about what co-design entails, highlighting the need for greater clarity (Cruickshank et al., 2013; Roper et al., 2018; Vargas et al., 2022). This is reflected in a recent commentary critiquing the inclusion of co-design methods within mental health research that involve people with lived experience in tokenistic or superficial ways (Colder Carras et al., 2023).

In mental health contexts, co-design has come to mean an approach in which people with lived experience of mental distress and mental health professionals and/or researchers combine their relative expertise to identify and prioritize problems and seek solutions (McKercher, 2021). Lived experience involvement and participatory approaches in research are currently stipulated as a requirement when applying for many research grants. For instance, the Australian Government’s primary health and medical research funding agency, the National Health and Medical Research Council (NHMRC, 2016), requires researchers to demonstrate their engagement with consumers and communities in grant applications to ensure their work addresses the needs of people experiencing the health conditions being researched. Lived experience involvement in mental health research is increasingly expected and enshrined in policy and research practices (Jones et al., 2021). This reflects the importance placed on engaging with consumers and the community in guiding the health and medical research sector on the meaningful engagement of consumers throughout all stages of research and health care.

Research in mental health has been critiqued for its inaccessibility and lack of relevance to consumers, acknowledging the need for consumers to be involved in setting research priorities (Banfield et al., 2018). Indeed, research priorities may be different for consumers, their families, and health practitioners. For example, Wyder et al. (2021) found that interest in research and breadth of priorities varied between consumers and health practitioners. This indicates that traditional health-driven research may not always address consumer needs, as researchers have not partnered with consumers to prioritize research questions that are meaningful and relevant to their needs. While increasingly recognized, co-design can be challenging to undertake meaningfully as it requires awareness and actions to balance power dynamics (Roper et al., 2018). The expertise of people with lived experience has historically been undervalued, and there is a need for co-design practices that incorporate safety and equity as integral components (Palmer et al., 2021, 2023).

In parallel with policy imperatives for greater consumer and community involvement, numerous co-design guides, publications, and toolkits have been developed (Bagley et al., 2016; Consumers Health Forum of Australia, 2017). However, little is available that outlines specific considerations and practices of co-design within mental health research. While previous scoping reviews have mapped the range of involvement of people with lived experience and the scope of collaborative research processes (Sangill et al., 2019), our review focused on exploring the understanding of co-design components and processes applied to research. The aim of this review was to identify and synthesize guidelines and models of co-design that have been applied within mental health research to understand how co-design is defined, the elements, values, and design tools that underpin co-design approaches and identify recommendations for the implementation of co-design. Clarifying these considerations will have practical value in operationalizing the critical factors of co-design and improving the consistency of its implementation in mental health research across a range of disciplines including psychology, nursing, social work, and occupational therapy.

## Method

A systematic scoping review methodology was used to map existing literature and address the aim. Scoping reviews synthesize existing literature, including both peer-reviewed and non-peer reviewed sources (Mak & Thomas, 2022). The Preferred Reporting Items for Systematic Reviews and Meta-Analyses extension for Scoping Reviews (PRISMA-ScR) was used as the reporting framework (Tricco et al., 2018) and provided in Supplementary Table 1. The research team included university academic/faculty members from various health-related disciplines, as well as researchers with personal experience in the topic, who bring lived experience perspectives to their work. The term “traditional academics” is adopted in the paper to refer to academics/faculty members and researchers with a background in health-related disciplines, as distinct from those employed as lived experience academics or researchers.

### Definition of Co-Design

The definition of co-design adopted in this review responds to criticisms from Colder Carras et al. (2023) about the superficiality or tokenistic involvement of lived experience in co-design. As such, co-design was defined as a participatory approach involving people with lived experience and researchers that explicitly outlines the roles and tasks of people with lived experience, including involvement in decision-making, addressing power imbalances and opportunities for collective empowerment. In addition, we acknowledged that co-design can form a discrete part of the research process rather than be used across the entirety of the research process (Roper et al., 2018).

### Inclusion and Exclusion Criteria

The *JBI population, concept, and context framework* for scoping reviews was used to define the inclusion criteria using a systematic approach to select studies aligned with the review’s objectives (Pollock et al., 2023). All studies that reported on co-design (or co-production or participatory action research [PAR] or patient and public involvement [PPI] or co-creation) approaches with lived experience in mental health research were included (Table 1).

**Table 1.** Scoping Review Inclusion Criteria.

PCC element	Definition
Participants	Literature involving co-design methodology that includes research conducted by people with lived experience of mental distress and/or carers together with health practitioners/researchers. Involvement in codesign required more than simply participating in research; it encompassed active engagement throughout the project.
Concept	Studies that discussed the application of co-design in research and included guidelines or frameworks that considered values and principles, processes, and tools. Terms used to describe similar participatory approaches were included, e.g., co-production, participatory action research, patient and public involvement, and co-creation.
Context	The literature was restricted to research using co-design research processes and methods within mental health settings and populations.

Studies were excluded if they were not undertaken in mental health settings and were not explicitly focused on research (e.g., education, service design, or service evaluation). There were no restrictions on countries and publication dates; only papers with full texts published in English were included.

### Search Strategy

An initial limited search of PubMed and CINAHL (EBSCOhost) was undertaken to identify a range of relevant articles. Keywords from titles and abstracts were identified to develop a comprehensive search strategy, incorporating terms related to mental health, research, and co-design with lived experience participants. The search strategy was adapted for each database. Five electronic databases, PubMed, Scopus, CINAHL, PsychINFO (Ovid), and Embase, were systematically searched for eligible peer-reviewed literature. Databases were searched from their inception to November 2023, when this study was initiated. A copy of the search process is provided in Supplementary 2.

The gray literature was identified through Google advanced searches using combinations of keywords that described co-design research within mental health settings. Google Advanced search queries allowed for more specific searches using exact phrases. Reference lists and citations of included articles and gray literature were manually searched for relevant studies not captured in initial search results. Database search results were exported to Covidence, and duplicates were removed. Two researchers, including a lived experience researcher and a health researcher (HR and VS), used the inclusion/exclusion criteria to screen titles and abstracts independently and then screened included full texts. Any discrepancies were discussed until a consensus was reached.

### Data Extraction

Data, including methodological features (e.g., study design, participants, settings), country of origin, definitions of co-design, reasons for co-design, co-design processes, underpinning principles, values and frameworks, facilitators/barriers, and outcomes/changes as a result of co-design, were extracted from the included studies into an Excel spreadsheet (Supplementary 3). For example, the concepts of underpinning principles, values, and frameworks were operationalized by identifying reported theories and guiding approaches to co-design within each study (when available). Data analysis compared information within each extraction category, highlighting commonalities and variations. After piloting 10% of the included references, one researcher (HR) extracted the information, discussing any concerns with the research team. A final quality check of the data extraction was undertaken by another researcher (VS). As common in scoping reviews (Peters et al., 2015), an appraisal of the quality of the studies was not undertaken.

### Data Analysis

Given the heterogeneity of the review results, a narrative synthesis was undertaken. The research questions provided a framework for analyzing the data, with a thematic analysis employed to identify recurring themes and patterns across the literature. Relationships, similarities, and differences in the data were then explored (Popay et al., 2017) by one researcher (HR). Both researchers (HR and VS) discussed emerging themes until a consensus was reached. The entire research team then reviewed these themes to ensure consensus.

## Results

**Figure 1.**
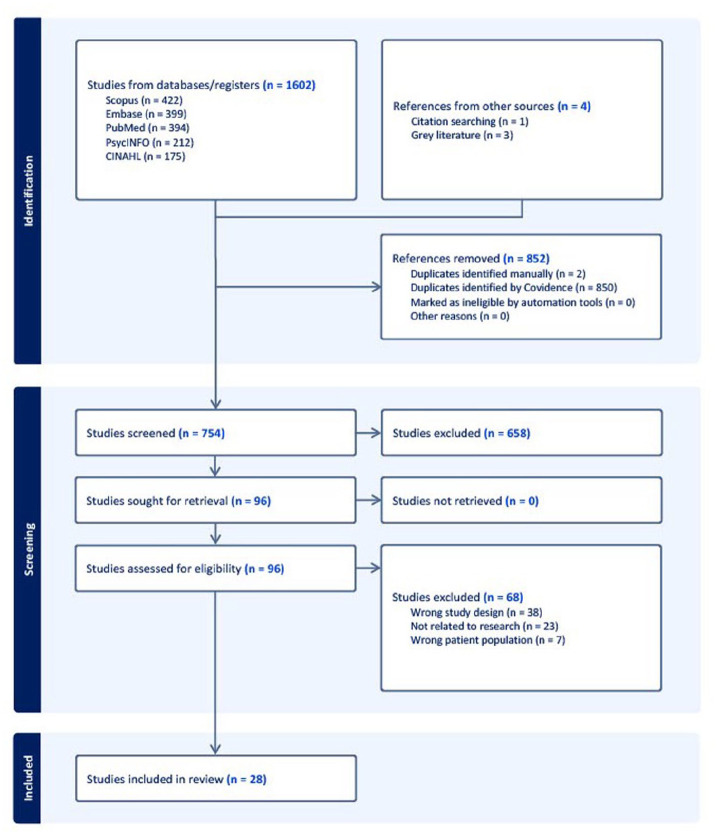
PRISMA Flow Diagram of Documents Identified in the Scoping Review.

Figure 1 outlines the search results in a PRISMA flow diagram. A total of 1,606 records were identified across all searches, and 852 duplicative records were removed. A total of 658 records were excluded during title and abstract screening, and 68 records were excluded during the full-text review. A total of 28 peer-reviewed texts and three gray literature documents met full eligibility criteria.

Included studies were published between 2012 and 2023 with most published in the last 5 years (*n* = 23). The majority of documents were published in the United Kingdom (*n* = 13) and Australia (*n* = 10), with one study each from Belgium, India, the Netherlands, New Zealand, and Sweden. Supplementary 3 provides an overview of the included literature.

The articles covered a wide range of topics, and the following broad themes around co-design were identified: (a) Delineating and defining co-design approaches, (b) Changes attributed to co-designed research, (c) Setting the parameters of co-design, (d) Learnings, and (e) Recommendations. The following sections provide further details regarding the five themes.

### Delineating and Defining Co-Design Approaches

The terminology varied greatly across the literature and was used inconsistently, interchangeably, or alongside other terms to describe different approaches. The most commonly used terms were Co-design, Patient and Public Involvement, and Co-production (Table 2).

**Table 2.** Terminology and Sources.

Terms used	Sources
Co-design	Bellingham et al., 2023; Palmer et al., 2023
Patient and Public Involvement (PPI)	Grundy et al., 2019; Worsley et al., 2021
Participatory Action Research (PAR)	Beeker et al., 2021; Haarmans et al., 2022; Miroy et al., 2022; Pillai et al., 2023; Richard et al., 2017; Sharmil et al., 2021
Co-creation	Fitzpatrick et al., 2023
Co-production	Bellingham et al., 2021; Faulkner et al., 2021; Gillard, Borschmann et al., 2012; Grim, 2022; Goldsmith et al., 2019; Kidd & Edwards, 2016; King & Gillard, 2019; Lambert & Carr, 2018; Machin et al., 2023; Pinfold et al., 2015; Rising Together Action Group, 2022; River et al., 2023; Roper et al., 2018; The PARTNERS2 writing collective; Worsley et al., 2021
Collaborative	Groot et al., 2022
Dialogical	Tomlinson & De Ruysscher, 2020
Service user involvement	Gillard, Borschmann et al., 2012

Co-design was generally defined using the levels of participation (e.g., Beeker et al., 2021 and River et al., 2023), whereas co-production emphasized partnerships and moving from “done on” to “done with” (Kidd & Edwards, 2016). Co-production was often viewed as a transformative approach considering power, resources, partnerships, risks, and outcomes (Lambert & Carr, 2018). PAR was described as a political approach, particularly useful in exploring inequalities, injustice, and marginalization and legitimizing a multiplicity of ways of knowing (Haarmans et al., 2022). Within First Nations research, a PAR approach was interwoven with specific Indigenous collaboration methods with strengths in developing feasible solutions (Sharmil et al., 2021). PPI terminology was primarily used in literature from the United Kingdom and focused on research processes governed by established standards informing inclusive opportunities, working together, support and learning, communications, impact, and governance (National Institute for Health and Care Research, 2019). Distinguishing lived experience involvement as distinct from public involvement was described as a challenge within PPI frameworks (Grundy et al., 2019; Machin et al., 2023; Worsley et al., 2021).

In an Australian First Nations study, PAR (Dadirri-Ganma methodology) focused on creating a bridge between non-Indigenous and Aboriginal and Torres Strait Islander knowledge. These included cycles of looking and listening, thinking and reflecting, collaborating and consulting, and planning and taking action (Sharmil et al., 2021).

#### Motivations for Co-Design in Research

The primary justifications for using participatory approaches in research were linked to:

- improving the quality and relevance of research (Bellingham et al., 2021; Gillard, Borschmann, et al., 2012; Goldsmith et al., 2019; Groot et al., 2022; Grundy et al., 2019; Kidd & Edwards, 2016; Lambert & Carr, 2018; Richard et al., 2017; Rising Together Action Group, 2022; Roper et al., 2018);
- creating ethical research (Groot et al., 2022; Haarmans et al., 2022; Richard et al., 2017; Roper et al., 2018; Sharmil et al., 2021; Tomlinson & De Ruysscher, 2020);
- guidelines for best practice and requirements for funding (Bellingham et al., 2023; King & Gillard, 2019; Machin et al., 2023; Worsley et al., 2021);
- focused on human rights, social and epistemic justice imperatives (River et al., 2023).

### Changes Attributed to Co-Designed Research

Different benefits of co-designed research were identified. These included identifying tangible outcomes of more relevant research and richer data, as well as intangible outcomes, which included changes in practice and relevant research questions as well as priorities from a lived experience perspective (Bellingham et al., 2021; Palmer et al., 2023; Worsley et al., 2021). The data collected was generally described as richer (Faulkner et al., 2021), and the distribution and uptake of knowledge were more substantial when compared with the standard outputs (River et al., 2023). The process developed by River and colleagues (2023) noted that codesign “led to delivering diverse outputs and outcomes that were relevant to both academic audiences and affected communities” (p. 6). Gillard, Simons et al. (2012) noted that co-design provided more nuanced findings that would otherwise have been missed. They reported that the involvement of lived experience researchers in the analysis explored interpretive perspectives of different research team members and retained the distinctive themes in their reporting. Here, the diverse perspectives of people with lived experience and health professionals provided a more complex analytic narrative. People involved in the co-design process found the findings more relevant for informing service improvements and change, claiming that findings “enabled a deeper discussion and identification of the different elements needed to restructure services” (Sharmil et al., 2021). Additional benefits included changes to research practices, including recruitment, implementation, and dissemination of findings and improved engagement with consumers and staff for conducting research (Richard et al., 2017).

### Setting the Parameters of Co-Design

Several theoretical frameworks underpinned the participatory research approaches. The most common for co-design were social learning theory, social constructivism, narrative methods (Palmer et al., 2023), critical theory, and standpoint epistemology (Goldsmith et al., 2019). Notably, PAR was considered to hold strong theoretical underpinnings and emphasized theories of social change made possible through social learning theory and liberatory approaches (Haarmans et al., 2022; Pillai et al., 2023; Richard et al., 2017). First Nations studies also drew on critical race theory and feminist inquiry (Haarmans et al., 2022; Sharmil et al., 2021). Other studies drew on peer support theories such as Intentional Peer support, critical consciousness, embodied connectedness (River et al., 2023), and personal recovery frameworks (Grim et al., 2022).

Despite the diversity in the theoretical frameworks, most studies shared similar values (also referred to as mindsets). All sources emphasized the importance of sharing power between academics and those with lived experience to ensure that Lived Experience is positioned as an expertise (Bellingham et al., 2021; Grundy et al., 2019; Roper et al., 2018). Given the inherent power imbalances within traditional research teams, some advocated for affirmative actions to ensure lived experience knowledge and expertise were privileged and able to influence decisions (Bellingham et al., 2021; Roper et al., 2018). Power sharing was also seen as critical in addressing epistemic injustice, as well as historical and current power inequalities (Bellingham et al., 2021; Grim et al., 2022; Roper et al., 2018). This was also an important consideration when setting the research agenda and determining who owns the findings/stories (Faulkner et al., 2021; Tomlinson & De Ruysscher, 2020).

For First Nations communities, social justice and power sharing were also linked to cultural safety, essential in creating holistic, ethically sound research approaches through respect, collaboration, active participation, and meeting the needs of communities (Sharmil et al., 2021). Cultural safety was also seen as important when researching ethnic cultural minorities (King & Gillard, 2019).

### Learnings

#### Barriers and Challenges

While co-design was seen as critical, many barriers were identified. One of the main challenges was working within the constraints of traditional research methods and institutions. Research hierarchies in academia were seen to replicate power imbalances experienced by those with mental health distress (Beeker et al., 2021; Haarmans et al., 2022; River et al., 2023; THe PARTNERS2 writing collective, 2020). Typically, narrow definitions of who could lead funded studies required a lead researcher with formal academic qualifications, representing a common bias that formal education is an indicator of competence, with other forms of knowledge, such as lived experience, being overlooked (Beeker et al., 2021; Lambert & Carr, 2018). It was also noted that many lived experience members of the research teams did not have the levels of research knowledge or an understanding of research language that enabled full participation (Gillard, Borschmann, et al., 2012; Machin et al., 2023). This presented barriers to the amount of lived experience involvement in decision-making and within research processes (Grim et al., 2022). These inherent power imbalances within academia have resulted in ongoing structural barriers to fully engaging in co-production, with traditional researchers maintaining ownership and control of decisions (Bellingham et al., 2021).

Other systemic barriers to co-designed research included grant funding priorities, short funding cycles, and insufficient training and employment opportunities for lived experience researchers (Grim et al., 2022; Machin et al., 2023; River et al., 2023). For academic researchers, barriers included time constraints and the labor-intensive requirements of co-design approaches, which were not factored into their workloads or funding guidelines (Haarmans et al., 2022; Kidd & Edwards, 2016; Machin et al., 2023; Pinfold et al., 2015; River et al., 2023; THe PARTNERS2 writing collective, 2020; Worsley et al., 2021).

Requirements imposed by some Human Research Ethics Committees were also seen as a barrier, with lived experience experts often viewed as a homogeneous group of people unable to provide consent (Lambert & Carr, 2018). In addition, traditional research methods were seen as having more power and credibility (Fitzpatrick et al., 2023). In particular, quantitative and randomized controlled trial (RCT) methodologies, which are characterized by strict and formal methodological criteria, posed challenges to the co-production scope (Goldsmith et al., 2019). Questions regarding the relevance and utility of traditional research approaches from a lived experience perspective were raised (Bellingham et al., 2021; Kidd & Edwards, 2016), with the notion of objectivity in any research method questioned (Bellingham et al., 2021). This seemed counterintuitive, considering that funding guidelines currently encourage co-design research; however, constraints precluded the adoption of methods that may have more effectively drawn on lived experience. A transdisciplinary approach and increasing reflexivity and social accountability of knowledge production offered opportunities to critique scientific approaches (Gillard, Borschmann, et al., 2012).

Binary terminology used to identify as lived experience or traditional researchers created challenges by not accounting for layers of experience and heterogeneity within research teams (Beeker et al., 2021). Distinctions in identity were not always clear, with members disclosing researcher, lived experience, or both identities over time. It was also recognized that creating a dichotomy between lived experience and traditional researchers may reproduce power differentials (Beeker et al., 2021), and that all involved bring a range of identities to research (e.g., gender, religion, culture). In fact, involving a range of identities in participatory research (THe PARTNERS2 writing collective, 2020), and challenging binary understandings of identity can facilitate reflections on assumptions that were made about others (THe PARTNERS2 writing collective, 2020).

Lived experience researchers described the subordination of lived experience knowledge, with reports of being silenced and feeling powerless (Bellingham et al., 2021). A lack of planned involvement, low expectations, and insufficient numbers of lived experience researchers were reported (Kidd & Edwards, 2016; Lambert & Carr, 2018). Working with emotions was identified as a potential challenge within participatory research (Machin et al., 2023; Palmer et al., 2023; Worsley et al., 2021). There were identified risks for lived experience researchers in using emotional labor (River et al., 2023) and for traditional researchers to be able to hear dissenting voices (Kidd & Edwards, 2016). Trauma, emotional safety, and the relevance of sharing were identified as challenges (THe PARTNERS2 writing collective, 2020; Worsley et al., 2021), with some recommending that attention be paid to ensuring lived experience researchers were adequately supported both emotionally and practically (Machin et al., 2023; THe PARTNERS2 writing collective, 2020). Teams needed to listen deeply to each other, sit with emotionally distressing information and balance individual issues with the collective needs of diverse groups (Sharmil et al., 2021).

#### Facilitators of Participatory Practices

Co-design was often described as intentionally relational, with the quality of relationships contributing significantly to the success of the co-design research process (Richard et al., 2017). It was acknowledged that relationship building depended on creating spaces to develop trust, facilitate discussion, and promote inclusion in decision-making (Palmer et al., 2023; Worsley et al., 2021). Communication was emphasized as crucial for respecting diverse knowledge, promoting open discussions, and bridging epistemic differences, which were seen as impossible without tension (Beeker et al., 2021). Creating a safe space requires being aware of power dynamics and providing opportunities to question and challenge one’s own power and personal biases. While participatory research often involved compromises and conflict (Grundy et al., 2019; THe PARTNERS2 writing collective, 2020; Worsley et al., 2021), maintaining connections within the team was seen as a fluid process, with a need for all to show care for each other (Groot et al., 2022). An aspect of creating safe spaces was reflected in the research team’s composition, including the number and percentage of lived experience researchers, as well as the experience and expertise they brought.

In addition, a First Nations study highlighted culturally appropriate processes such as sharing food, traveling to Country, and allowing time for yarning and rapport-supported relationship building as important participatory practices (Milroy et al., 2022). Ensuring people were heard and respectful ways of being together were identified as contributing to the research’s success. The strategies could be grouped as: (a) continual effort was needed to build and maintain relationships and communication (THe PARTNERS2 writing collective, 2020; Tomlinson & De Ruysscher, 2020); (b) practices of sharing power and resources helped position lived experience as legitimate researchers, and connected them to the research team (Haarmans et al., 2022; River et al., 2023; Roper et al., 2018). The strategies used for these practices were varied and are described in detail in Table 3.

**Table 3.** Recommended Practices to Support Relationships and Share Power.

Practices supporting relationships	
Continual effort needed to build and maintain relationships and communication (THe Partners2 Writing Collective, 2020; Tomlinson & De Ruysscher, 2020).	Structuring meetings through rules, rituals and routines (i.e., check-in, rotating roles, traffic light system to support safety) (Beeker et al., 2021; Haarmans et al., 2022) Having both larger and smaller group discussions to provide input and balance diversity to address power symmetries (Grundy et al., 2019; Milroy et al., 2022)
	Willingness to engage in difficult conversations and have disagreements (Worsley et al., 2021) Ethics of relational safety (Richard et al., 2017) Open communication to build trust (Beeker et al., 2021; Machin et al., 2023; THe PARTNERS2 writing collective, 2020)
	Slowing down decisions and taking turns speaking to stop voices being silenced (Kidd & Edwards, 2016) Reviewing the group agreement, using a talking stick and showing the importance of being attentive to interpersonal dynamics within the team(Haarmans et al., 2022)
	A joint work plan from the outset, discuss skills, roles and leadership. Who is doing ethics, liaising, reporting, organizing, for example, travel, recruitment, designing, and implementing dissemination (Kidd & Edwards, 2016)
	Making space for the sharing of personal information (Haarmans et al., 2022)
	Explicit valuing of lived experience and being open to new ideas and diversity (Grundy et al., 2019)
	Beginning the process with “healing sessions” where trusted relationships are afforded time to develop may reduce negative impacts and enable the process to deliver more benefits than costs. As tensions come in and out, “healing sessions” could also be scheduled responsively throughout the process (Worsley et al., 2021)
Practices supporting the sharing of power	
Practices of sharing power and resources helped position lived experience as legitimate researchers and connected to the research team (Haarmans et al., 2022; River et al., 2023; Roper et al., 2018).	Deciding on acceptable language was seen as helpful (Haarmans et al., 2022; Roper et al., 2018)
	Power-sharing involved explicitly acknowledging different experiential, ideological and epistemological positions within the team and visible ways that differences in expertise are included and valued (THe PARTNERS2 writing collective, 2020)
	Power is inherent in research hierarchies, and it was vital to name this power and actively share and relinquish power (Beeker et al., 2021; THe PARTNERS2 writing collective, 2020) Being open about structural inequalities, how we hold power, and, how inaction (not using power) can influence decisions was important (Haarmans et al., 2022)
	Transparency in decision-making is needed (Beeker et al., 2021; THe PARTNERS2 writing collective, 2020) Clarify decision making processes—is it unanimous or majority? Whose voices are prioritized? (Kidd & Edwards, 2016)
	As part of creating epistemic justice, Bellingham and colleagues recognized the role of equipping lived experience researchers with training and skills to work effectively within research settings. Training improves the research knowledge of people with lived experience and can provide them with the hermeneutical resources to make sense of research practices and power relations (Bellingham et al., 2021)

### Recommendations

Many studies offered recommendations to support others’ endeavors in participatory research. Most recommendations were based on reflections and experiences, often reflecting the identified values, principles, and practices as ways of overcoming challenges and capitalizing on facilitating factors. Ethical approaches that were able to incorporate a level of nuance when considering vulnerability were recommended to prevent the exclusion of participation partners (Lambert & Carr, 2018). In fact, participatory approaches were seen as a way to engage with marginalized groups rarely represented in research, providing appropriate support for engagement at a range of levels (Lambert & Carr, 2018).

For co-design in research to succeed, experiential knowledge needed to be recognized by all parties as a fully valued form of knowledge (Tomlinson & De Ruysscher, 2020). In addition, allowing space for critiquing and challenging dominant ways of knowing about health and health research was recommended (Goldsmith et al., 2019). The Rising Together report (Rising Together Action Group, 2022) discussed the role of the provocateur, or curious questioner, as part of the co-design process. The role of this team member allowed for questioning of assumptions and unspoken understandings, and explored processes and findings.

Identified recommendations are presented in Table 4 and described in connection with different phases of research.

**Table 4.** Participatory Research Recommendations.

Research phase	Recommendation
Before undertaking participatory research	Ensure team values and culture that support co-design, including valuing lived experience perspectives and relationships that recognize dynamics of power are established (Goldsmith et al., 2019; Tomlinson & De Ruysscher, 2020; Worsley et al., 2021)
	Lived experience researchers warn against half-hearted commitment, stressing that everyone involved in participatory research needs to be fully onboard (Roper et al., 2018)
	Leaders and decision-makers must understand and support the co-design process before it starts. Supports capability and confidence, including knowledge of research processes and language (THe PARTNERS2 writing collective, 2020)
	Ensure the funding supports co-design approaches including adequate reimbursement (Grundy et al., 2019) Worsley et al. recommended a separate participatory research funding stream (Worsley et al., 2021)
	Training for team members (lived experience and academics) to navigate epistemic differences and power asymmetries (Bellingham et al., 2021) This includes attention to building confidence and reflexivity, sharing skills, being conscious of “us and them” divisions, how disclosures are made and what is reflected by job titles (Machin et al., 2023; THe PARTNERS2 writing collective, 2020)
	Clear roles and leadership plans are in place (Kidd & Edwards, 2016)
	Build in mechanism for evaluation and feedback (Machin et al., 2023)
During participatory research	Clear underpinning model of co-design and understanding of approaches across the research process (Tomlinson & De Ruysscher, 2020)
	Invest in relationships and ensure team commitment to a dialogical research process (Tomlinson & De Ruysscher, 2020) This can include slowing down the process to enable shared decision-making and healing work for lived experience researchers to build trust and reduce the negative impact of past trauma (Worsley et al., 2021)
	Co-authoring is a precious relational process and epistemic diversity needs to be included (Groot et al., 2022)
	Co-learning space supported, enables traditional researchers to share knowledge and resources for navigating research cultures and systems that are often unfavorable to participatory practice (River et al., 2023)
	Support flexible and non-linear approaches to research (Tomlinson & De Ruysscher, 2020)
	Build in support processes, for example, peer reflection sessions (Machin et al., 2023)

## Discussion

With an increasing prioritization of lived experience engagement in research, this scoping review aimed to explore co-design elements, steps, and processes that have been applied within mental health research contexts. While not all studies were underpinned by specific models or frameworks, several were described. Some researchers had developed their own models: (a) Living Labs (Palmer et al., 2023) who reported a model and components outlining steps and critical elements and (b) Raising the Bar (River et al., 2023) which was a co-developed approach to ensure a high level of involvement and equitable decision-making and power throughout all stages of the research, including planning, co-design, conducting, and co-dissemination. Similarly, the Rising Together project outlined “scaffolding” in ways of being together and bringing the group into connection (Rising Together Action Group, 2022). Researchers also drew on existing models, such as the Four Ps framework comprising principles, purpose, presence, and power (Machin et al., 2023). Other approaches included bricolage as theoretical and practical guidance for the research process (Tomlinson & De Ruysscher, 2020), a relational engagement model (Richard et al., 2017), and a radical reflective approach to considering the extent coproduction was used (Gillard, Simons, et al., 2012).

Many values and principles were common across the included studies, such as investing in relationships and power-sharing processes. While researchers varied in their understanding and privileging of lived experience, all sources acknowledged a need to shift existing mindsets and establish a culture that genuinely honored and legitimized people with lived experience as experts. Roper et al. (2018, p. 5) stressed that co-design “privileges consumer perspective, and promotes and develops consumer leadership, which shifts away from a historical positioning of ‘professionals’ as the experts that steer the agenda.” Researchers with lived experience needed to be seen as partners with valued knowledge and expertise that was actively sought to inform traditional research practices and processes (Haarmans et al., 2022; THe PARTNERS2 writing collective, 2020; Worsley et al., 2021).

However, the difficulties of terminology and the definition of co-design were problematic, resulting in lived experience involvement in research being conflated with participation more broadly (Grim et al., 2022). The inclusion of lived experience was also recognized as a challenge in relation to binary identities, resulting in confusion about which part of one’s identity was foregrounded in different research situations. There was confusion about lived experience researchers, researchers with lived experience, and lived experience more broadly, with traditional researchers questioning the best way to integrate lived experience perspectives in a meaningful way. The literature also identified issues regarding representation of lived experience, specifically what *experience* is most relevant to the research context, how to ensure diversity and supports necessary to involve those often excluded from processes.

Traditional research methods, inherent hierarchies, and structures affected co-design. Despite co-design being an imperative, little has been done to address these issues with current grant processes, timing, resourcing, journal requirements, ethics approval processes, and so on, resulting in different valuations of knowledge/expertise and inequities within research teams. Our review found that often, these processes restricted research methods and steps that were seen as more appropriate to codesign. Innovative and creative approaches, such as arts-based or digital stories, were seen as important in improving inclusivity, engagement, and empowerment for people from marginalized backgrounds (Pillai et al., 2023). Importantly, ensuring adequate time and resources to undertake co-design within research was identified as a significant barrier (Lambert & Carr, 2018; River et al., 2023). Recommendations emphasized the need to advocate for addressing structural barriers and incorporating a social justice lens to promote openness, learning, and cultural change within research environments.

In addition, the contribution of lived experience roles and the emotional labor needed for many to participate in research needs to be recognized and appropriate support provided. An important consideration is the precarity of working conditions, with many people within identified lived experience roles employed casually and remunerated disproportionately to traditional researchers. While this may reflect current research funding arrangements, it suggests that the contribution of lived experience is of less value and perpetuates existing power imbalances. Our findings indicated that co-design requires additional emotional resources from all team members (e.g., managing tensions and conflict, hearing distressing experiences, and maintaining relationships). The importance of safety for members of the research team was identified as critical. Without safety, it was hard for all to feel included and express their ideas. The need for safety and attention to the role of power was a key difference in working with historically oppressed and marginalized groups, such as those with lived experience of mental distress (Mulvale et al., 2019).

The review also gathered practical guidance and recommendations for engaging in ethical co-design. However, it was recommended that researchers not rely on standardized approaches to co-design, emphasizing the need for flexibility and responsiveness within different contexts. In line with warnings from Colder Carrass (2023), against publishing tokenistic research and calling it “participatory,” this review also identified risks in inappropriate or misleading use of codesign and potential harms from co-optation. This is particularly important in mental health research, with key recommendations from the literature advocating for addressing issues of power and ensuring that power imbalances that sustain epistemic injustices are not perpetuated or made worse. The review also shared learnings from research exploring co-design and offers guidance regarding how co-design can stay true to the intention of creating equal partnerships in mental health research.

## Strengths and Limitations

The findings from this review need to be considered within the context of its strengths and limitations. This review adhered to the PRISMA guidelines, and quality checking of screening and data extraction, along with a team approach to theming the documents, added transparency and rigor to the research. The research team consisted of lived experience academics and traditional academics, allowing for reflexivity from different perspectives when theming the data. A notable strength and limitation of this review was the diverse range of studies examined, including both academic and gray literature. This variety provided valuable insights into how co-design is operationalized in different settings. However, the heterogeneity of the studies prevented direct comparison. This may reflect the diversity in co-design implementation in mental health research. In addition, the heterogeneity of the studies made it challenging to apply a uniform approach to data extraction, as descriptions of co-design practices were often ambiguous, lacking in detail and used diverse terminologies.

## Conclusion

Despite increasing calls and identified advantages for co-design approaches in mental health research, integrating co-design into usual research practices continues to be a challenge. This scoping review explored co-design processes, identifying essential components and steps that support co-design in mental health research. Key barriers included traditional academic research structures, such as inflexible funding mechanisms and hierarchical decision-making. However, the review also highlighted opportunities for innovative research approaches and outcomes. The study provides examples of processes to address these barriers and offers practical recommendations for operationalizing co-design in mental health research. It emphasizes the need for a shift in mindsets to genuinely honor and legitimize people with lived experience as experts, and the importance of addressing structural barriers to ensure inclusivity, engagement, and empowerment.

## Supplemental Material

sj-docx-1-otj-10.1177_15394492251367259 – Supplemental material for Scoping Review of Co-Design in Mental Health Research: Essential Elements and Recommendations Supplemental material, sj-docx-1-otj-10.1177_15394492251367259 for Scoping Review of Co-Design in Mental Health Research: Essential Elements and Recommendations by Helena Roennfeldt, Victoria Stewart, Marianne Wyder, Teresa Fawcett, Caroline Robertson, Rebecca Soole, Dan Siskind, Amanda Wheeler and Justin Chapman in OTJR: Occupational Therapy Journal of Research

sj-docx-2-otj-10.1177_15394492251367259 – Supplemental material for Scoping Review of Co-Design in Mental Health Research: Essential Elements and Recommendations Supplemental material, sj-docx-2-otj-10.1177_15394492251367259 for Scoping Review of Co-Design in Mental Health Research: Essential Elements and Recommendations by Helena Roennfeldt, Victoria Stewart, Marianne Wyder, Teresa Fawcett, Caroline Robertson, Rebecca Soole, Dan Siskind, Amanda Wheeler and Justin Chapman in OTJR: Occupational Therapy Journal of Research

sj-docx-3-otj-10.1177_15394492251367259 – Supplemental material for Scoping Review of Co-Design in Mental Health Research: Essential Elements and Recommendations Supplemental material, sj-docx-3-otj-10.1177_15394492251367259 for Scoping Review of Co-Design in Mental Health Research: Essential Elements and Recommendations by Helena Roennfeldt, Victoria Stewart, Marianne Wyder, Teresa Fawcett, Caroline Robertson, Rebecca Soole, Dan Siskind, Amanda Wheeler and Justin Chapman in OTJR: Occupational Therapy Journal of Research
