# Supplementary material for: Scoping Review of Co-Design in Mental Health Research: Essential Elements and Recommendations
Source: OTJR (Thorofare N J). 2025 Sep 8;46(2):311–23. doi: 10.1177/15394492251367259 (PMC12987994; doi:10.1177/15394492251367259)
Supplement: sj-docx-2-otj-10.1177_15394492251367259 – Supplemental material for Scoping Review of Co-Design in Mental Health Research: Essential Elements and Recommendations [file sj-docx-2-otj-10.1177_15394492251367259.docx]

Supplementary 2.

*Search strategy example*

| **CINAHL (EBSCOhost)** | | |
| --- | --- | --- |
| [**Search ID#**](javascript:__doPostBack('ctl00$ctl00$MainContentArea$MainContentArea$historyControl$ReorderHistoryLink','')) | | **Search Terms** |
|  | S20 | S14 and S18 and S19 |
|  | S19 | S15 or S16 |
|  | S18 | S17 and S13 |
|  | S17 | S11 or S12 |
|  | S16 | AB research* |
|  | S15 | MW participatory research |
|  | S14 | AB ("co produc*" or "co design*" or coproduc* or codesign*) |
|  | S13 | AB ("lived experienc*" or consumer* OR "service user* or peer) |
|  | S12 | AB ("mental health" or "mental ill*") |
|  | S11 | (MM "Mental Health") |
|  | S10 | s4 and s8 and s9 |
|  | S9 | s5 or s6 |
|  | S8 | s7 and s3 |
|  | S7 | S1 or s2 |
|  | S6 | AB research* |
|  | S5 | MW participatory research |
|  | S4 | AB ("co produc*" or "co design*" or coproduc* or codesign*) |
|  | S3 | AB ("lived experienc*" or consumer* OR "service user* or peer) |
|  | S2 | AB ("mental health" or "mental ill*") |
|  | S1 | (MM "Mental Health") |
