# Supplementary material for: Scoping Review of Co-Design in Mental Health Research: Essential Elements and Recommendations
Source: OTJR (Thorofare N J). 2025 Sep 8;46(2):311–23. doi: 10.1177/15394492251367259 (PMC12987994; doi:10.1177/15394492251367259)
Supplement: sj-docx-3-otj-10.1177_15394492251367259 – Supplemental material for Scoping Review of Co-Design in Mental Health Research: Essential Elements and Recommendations [file sj-docx-3-otj-10.1177_15394492251367259.docx]

Supplementary 3.

*Data extraction table*

| **Title / Date** | **Authors** | **Geographic location** | **Methodology/ Purpose** | **Population- diagnosis, culture, other intersectionality** | **Stakeholders/staffing** | **Research area/methods** | **Definition of Co-design** | **Why co-design?** | **Steps/process/model** | **Principles and Values & Ethics** | **Facilitators** | **Outcomes/ Change as a result of codesign** | **Barriers** | **Phase of research** | **Recommendations/ learning** |
| --- | --- | --- | --- | --- | --- | --- | --- | --- | --- | --- | --- | --- | --- | --- | --- |
| **Rising Together report / 2022** | Rising Together Action Group (2022). | Australia | The study sought to investigate the experiences of family/carer lived experience (LE) workers within the Victorian mental health system, with the aim of better understanding what is needed to ensure the safe and sustainable development of this workforce. | Co-designed research into the carer workforce in Victoria, Australia | Carers, consumers and conventional researchers. Role of the provocateur | Survey and photovoice | Uses Roper et al definition and model of coproduction | Looked at historical underpinnings and codesign as making sense and yielding best outcomes | Includes ways of being together and bringing the group into connection | Informed by the principles and frameworks outlined within Co-production: Putting Principles into practice in mental health contexts (Roper, Grey, and Cadogan, 2018), with adjustments made to fit a family/carer LE perspective. | Relationships | Reflections regarding some personal outcomes from the co-design process included e.g., confidence building | Limited detail re. co-design barriers provided. | Across all phases of the research |  |
| **Designed to Clash? Reflecting on the Practical, Personal, and Structural Challenges of Collaborative Research in Psychiatry / 2021** | Beeker et al. | UK | This paper provides an in-depth description of a three-year collaborative project that took place in the wider context of a mixed-method process evaluation of innovative models of psychiatric care in Germany. | Psychcare- comparative evaluation of psychiatric hospitals | People with LE in research roles | Mixed methods study. Interactive interviewing on research process. Reflections on research process | Collaborative and participatory research | Policy and service imperitive | Includes process and key points for doing collaborative research | Mutual trust and respect | Supervision, rituals, embracing or allowing conflict, communication | Not reported | Binary identities, clinical settings, accessibility, challenges with travel and requirements of study, structural power imbalances, language | Across all phases of the research | Routines and rituals for group meetings, fluid roles, breaks and signalling emotions and level of comfort, tandem or small group/pairs work, real change does not happen withut conflict. |
| **Towards epistemic justice doing: Examining theexperiences and shifts in knowledge of livedexperience researchers over the course of amental health research training programme / 2021** | Bellingham et al. | Australia |  | LE involvement in research | LE researchers and conventional researchers | Research -training for LE researchers | Within broader context of coproduction | Health service reform, human rights and social and epistemic justice. Relevant research priorities | evaluated training and specific requirements of people with LE. Longitudinal and qualitative study. Levels of participation and reference to Arnstein's ladder | Informed by theories feminist theories, Frierre, Mad Studies, Power sharing. Coproduction privelegs LE |  | Shifts in knowledge | Research domain is seen as the domain of conventional researchers, traditonal understanding of participation levels | Coproduction -co design, co deliver, co evelaute, Codeveloped and cofaciliated by LE researchers and conventional researchers |  |
| **Co-design Kickstarter-A short guide to get started, and become more familiar with co-design research / 2023** | Bellingham et al. 2023. https://cmhdaresearchnetwork.com.au/resource/co-design-kickstarter/ | Australia | Designed to support meaningful co-design in research | mental health and alcohol and other drugs sectors | This resource is for people who have an interest in deepening participation and co-designing research in ‘mental health’ and ‘alcohol and other drugs’, | The Co-design Kickstarter provides a guide to the co-design of research (co-design research) | Distinct from coproduction and exists as a continuum model of co-design | Aligns with calls from social and human rights movements, including Consumer/Survivor, Peer, and Disability movements (lived experience community) that have advocated for re-balancing and restoration of power in the production of knowledge. | Includes levels of research participation. Identifies codesign research activities within substantive codesign. | Includes need for epistemic justice | Facilitators identified for each phase of research. | Limited information | Focused on how to do co-design. | Guides for all phases or involvement in key areas | Specific to research. Continuum of co-design. Provides a clear conceptual understanding of co-design. Has a model of co-design and offers recommendations. |
| **‘Dignity and respect’: An example of service user leadership and co‐production in mental health research / 2021** | Faulkner et al. | UK | Use of codesign to address gaps on knowledge | Service users experiences of crime | The research team consisted of survivor researchers and aca‐ demics with practice experience. The principal investigator (SC) is a  survivor researchercollaboration with academics and professionals and SU | Elements of research were user-led. Reflections on processThe study was designed to create both real and virtual conversations  between service users, practitioners, policymakers and academics.  In order to achieve this, we designed a series of interconnected work  streams utilizing different data collection methods to facilitate dis‐ cussion | Co‐production means researchers, practitioners and 'members of the public' working together, sharing power and responsibility from the start to the end of the project, including the generation of knowledge. However, co‐produced research generally remains in the leadership of academic researchers. Our  understanding of a co‐productive approach to research involves collaboration between service users and practitioner and academic allies to transform the potential of research to achieve meaningful change. In this project, the principal investigator was a service user researcher and the study was co‐produced with practitioner academics and survivor researchers; hence our use of both terms 'user-led and co‐produced'. | Transformation of power relations, in a similar way to survivor or emancipatory research | The study was designed to create both real and virtual conversations between service users, practitioners, policymakers and academics utilising different data collection methods. | Control was given as much as possible to participants. Consent was never assumed. | All researchers in the team had group and peer supervision, in the form of reflective research practice, and had opportunities for post‐interview debriefing. There was sufficient flexibility in the budget to allow for overnight stays, a meal with a supportive friend or accessible transport if the researcher needed them following a distressing interview. The research team reviewed the ethical conduct of the research on an ongoing basis, using survivor research ethical principles as guidance. | Richer data | Histories of trauma and abuse - powerlessness and disengagement. | Across most phases of research | Continuous knowledge exchange |
| **Co‐ideation and co‐design in co‐creation research: Reflections from the ‘Co‐Creating Safe Spaces’ project / 2023** | Fitzpatrick et al., | Australia | Examine the utility of a publised systematic framework | Suicidality | People with lived experience of emotional distress and/or suicidal crisis, including academic researchers, service and peer workers, carers and advocates were involved in the co‐ideation and co‐design of this research. All authors identify as people with lived experience, from both academic and nonresearch backgrounds | Instrumental case study approach, we examined the utility of a published systematic framework designed to improve clarity about co‐creation as a concept and approach. The framework is explored based on the first two processes that correspond to our own work to date: co‐ideation and co‐design | Concept of co‐creation has typically been defined in one of two ways: (i) as the collaborative generation of new knowledge, or (ii) as the planning, implementation and evaluation of new services and programs. They contend that a complementary framework that acknowledges and incorporates both aspects is key to the concept of co‐creation and its capacity to address problems of collaborative involvement and power that can result if there is no requirement for collaboration | Benefits to design and delivery of services | Between service users, practitioners, policymakers and academics. | The values and principles identified in the initial co‐design of the safe spaces. These include respect, inclusion, choice, transparency, safety, lived experience‐led and valuing each person's experience and expertise. | Collective leadership, power sharing, safety and trust are essential | Novel survey question types, clarity re. language | While it is useful to think about what is involved in co‐ creation, it is difficult to pin down these processes | Used a case study approach, to explore the utility of the framework by describing two of these categories in our current co‐creation research project: co‐ideation and co‐design. | Co‐creation is messy and complex with much of the work invisible and dependent on relationships and shared values. Any sustainable outcome is therefore dependent on interpersonal relationships and the extent to which stakeholders, including researchers, establish and maintain genuine partnerships with each other. |
| **Legitimizing user knowledge in mental health services: Epistemic (in)justice and barriers to knowledge integration / 2022** | Grim | Sweden | To explore the barriers and facilitators to the legitimacy of user knowledge, as a central factor in sustainably implementing user influence in mental health practice. | Team included researchers and representatives from service user organisations | 6 researchers from various disciplines and 7 service user researchers | Explore barriers and facilitators of LE knowledgeUtilizing a co-production design, the team developed an interview framework and conducted a series of digital workshops | Theoretical foundation of epistemic injustice | address epistemic injustice | In order to achieve this, we designed a series of interconnected work | Model of creating justice in sharing different forms of knowledge | Precursors to effective co‐creation research | Potential for quality improvement | ideas of professionalisation and risk of cooption. Precarity in funding and employment. Self stigma and lack of confidence | Not clearly articulated |  |
| **Producing different analytical narratives, coproducing integrated analytical narrative: a qualitative study of UK detained mental health patient experience involving service user researcher / 2012** | Gillard et al. | UK | Explore the impact on research findings of involving service users in qualitative data analysis. | Detained mental health patient experiences | Service users and researchers. Our research team consisted of a professor of mental health nursing, two non-clinical, post-doctoral health services researchers and three part-time service user researchers with some previous experience in research, all employed by a London university medical school. | Semi structured interviews. narratives | Bringing together SU and researchers to coproduce knowledge | The potential to explore the impact on research findings of service user researchers’ involvement in conducting qualitative interviews, as well as analysing qualitative data, was suggested above. In order to allow an adequately detailed investigation this paper will focus on just one aspect of that involvement, analysis, where the impact appeared to be strongest. | Limited desciption of co-design process | Not outlined | not described | Service user researchers identified distinct themes during analysis. The points of contact in those three interpretations provided us with the means toadvance a more complex analytical narrative on the lived experience of detained care that integrated our differently situated analyses, as service users, health services researchers and nursing researcher. | Not described | Coproduce analytical narratives. Involved in development o questions, data collection, analysis strategy and analysus |  |
| **Patient and public involvement in the coproduction of knowledge: Reflection on the analysis of qualitative data in a mental health study / 2012** | Gillard et al. | UK | Describe co-design process and critically reflect on process. | 120 users of mental health services | Research team of 17 individuals comprised health service researchers (n = 6), a human resource management researcher, service user (patient) and carer (family or friend caregiver) researchers (n = 7), clinicians (n = 4), and National Health Service (NHS; U.K. statutory health managers (n = 2). | Mixed Methods. team process of qualitative data analysis undertaken in a study of support for self-care in mental health | Distinguishes coproduction from PAR- PAR creates action or change whereas coproduction within research is about contributing to new knowledge | To produce good evidence | Detailed description of analysis process | Radical reflective approach to consider the extent coproduction was used. | An essential component of how we coproduced knowledge involved retaining methodological flexibility so that nonconventional research voices in the team could situate and critique what was conventionally known. Deliberate and transparent reflection on how “who we are” informed the knowledge we produced was integral to our inquiry. Methodological flexibility | Team members not from research backgrounds sometimes challenged academic conventions, leading to complex findings that would otherwise have been missing. | Not described | Analysis | We conclude that reflecting on knowledge (co)production is a useful tool for evaluating the impact of patient and public involvement on health research. Not about bringing together knowledges but holding them as both valuable |
| **Co-producing Randomized Controlled Trials: How Do We Work Together? / 2019** | Goldsmith et al. | UK | To report the possibilities and challenges of coproducing a randomised controlled trial. | Research on multisite RCT of peer support in community mental health service | Diverse research team including 2 LE researchers | Methodological rigidity of RCT | In terms of a knowledge framework. Definition is provided based on work of Gilliard 2012. 6 elements- shared decision making, value different knowledge, involvement throughout research, flexibility, critical reflection and outputs that report on how knowledge was produced. | Nothing about us without us and good quality research | Decision making process | Underpinning frameworks standpoint epistimologies, critical disciplines | Establishing clearly what decisions and processes can be codesigned. Space for dialogue, reflection and creating culture | Potential use of co-design in RCT | Methodological rigidity of RCT | Front loaded at start to make sure involved in decsision making | Our findings suggested that, in a randomized controlled trial, the methodology demands that co-production is front-loaded wherever possible as it could be challenging for service user researcher members of the team to implement some research decisions into practice where they had not been involved in early decision-making. This means most of the time for co-production must be scheduled toward the start of the project. However, we also found that co-production of the trial analysis strategy worked well within circumscribed and well-communicated limits. |
| **Relational, ethically sound co-production in mental health care research: epistemic injustice and the need for an ethics of care / 2020** | Groot, Haveman & Abma | Netherlands | Collaborative reflection | Good care in crisis study aimed to improve emergency care | Traditional researchers, service users and mental health professionals. Also involved police and ambulance. Service users were accessing an acute crisis service | Collaborative reflection on process of coproduction. Participatory Health Research- power sharing | Values of emancipatory practice and giving voice to SU | Epistemological value- respect for diverse knowledge, practical and experiential elements. | Not described |  |  |  |  |  | Identified three critical moments: reporting; discussing the findings, reception, rewriting and reconnecting. Decisions made in the interests of time and meeting deadlines. Indicates who has power- who decided what gets written. Co authoring is a precious relational process and epistemic diversity needs to be included. Concerns about including creative mediums and being seen as unscientific. SU researchers still seen as SU. A lack of trust and fear of the emotions of SU. Care for both SU and professionals. Concerns about dissemination findings due to being too critical and damaging relationships. Maintaining connection within the team is challenging and fluid |
| **Public involvement in health outcomes research: lessons learnt from the development of the recovering quality of life (ReQoL) measures / 2019** | Grundy et al. | UK | Provide an example of patient involvement in the development of an outcome measure | Mental health service users | Service users, researchers, clinicians and stakeholders. Scientific group and expert service user group | Qualitative design. Governance model which involved SU alongside researchers and clinicians. Also advisory groups, Figure shows SU involvement in stages of research | Use definition of PI “research carried out ‘with’ or ‘by’ members of the public rather than ‘to’, ‘about’ or ‘for’ them” | Better outcomes and more relevant research | Outlines stages of codesign and SU involvement across these | Values of PPI- research without PI is irrelevant and of little worth | Expert service users met independently, all briefed appropriately, time and effort, processes to resolve disagreements | Measures were more meaningful, increased face and content validity of the measure. | Power asymmetry | Data analysis, interview, dissemination strategies | imperative that LE is involved from the outset and all stages. This meant data could be critiqued by LE throughout the research. Open to including new information at all stages. Nothing was set. Involving SU from inside and outside academia to minimise jargon and assumed knowledge. Welcome different perspectives and priorities. Bringing concerns about the emotional impact of the research. Welcoming disagreements. Having core values that you can refer to, particularly during disagreements. Addressing power symmetries by having both larger and smaller group discussions to provide input. Adequate reimbursement. |
| **The practice of participatory action research: Complicity, power and prestige in dialogue with the 'racialised mad'. / 2022** | Haarmans et al. | UK | Reflection on co-designed project | Minority SU- racialised mad | Ethnic minority LE researchers and traditional researchers. | Qualitative study exploring ethnic inequalities in mental health. Drawing on Foucault’s notion of power as relational, we focus on three key aspects of productive power: (1) relational engagement and reciprocity, (2) positioning coresearchers as authentic researchers and (3) adopting an ethic ofcare, to explore complicity and resistance in reproducing hierarchies of knowledge and power when attempting to create and sustain a PAR process for collective analysis, action and solidarity. We utilise retrospective and recorded reflections over the course of the project. | PAR. Heavily describes the role of power and history and theoretical underpinnings of PAR | Promote inclusive knowledge production. | Processes involved in relational ways of working, checking in, sharing personal inormation | Principles of PAR. Relational engagement and recipriocity. Mutual sharing within the team. Resisting power structures and sustaining solidarity |  |  | Not knowing how to respond to emotions and people feeling silenced or unsafe. Structural power relations | All phases | Use of the talking stick, checking in, sharing personal information and actively challenging power structures |
| **Doing it together: a story from the co-production field / 2016** | Kidd & Edwards | NZ | Examine the experiences of working in a co-produced research project | Research project on supported housing involving SU. Research team academics, LE researchers and managers | Research team- academics, LE researchers, managers. People receiving supported housing | Reflections on working on a research project. Research project on supported housing. Research began in 2010. Qualitative | Co-production in the context of mental health research has become something of a buzzword to indicate a project where mental health service users and academics are in a research partnership. Idea that many perspectives are better than one. | Not clearly described | Limited theory and process explaining co-production | Not clearly described |  |  | challenges with decision making roles and making decisions collectively given workloads, Fluid role identities. | All phases | Need for a leader to drive the project. Openness to wide array of experience that could enhance project and relationships. Did not value voices of SU. Slow down decisions, take turns speaking, notice when people were silent. Learning from mistakes. Need for a framework |
| **Bringing together coproduction and community participatory research approaches: Using first person reflective narrative to explore coproduction and community involvement in mental health research / 2019** | King & Gillard | UK | Reflection on coproduction process | Evaluation of a primary care mental health service | LE researcher, health researcher and team of community co-researchers. Included marginalised service users. | Evaluation of a primary health service. Cycle of reflective first person accounts. Traditional academic and service user and community researchers, including through service user/survivor leadership. | Hybrid coproduction and participatory approach | Part of the requirement for the evaluation | Narrative reflections on process/steps | Not clearly described |  |  |  | Coresearchers were involved in developing survey, interview and focus group tools, interviewing and conducting focus groups, analysing survey, interview and focus group data, writing up sections of the evaluation report and presenting findings at the evaluation report launch. | Cultural issues and need to feel safe. Need to create a welcoming space |
| **‘Outside the Original Remit’: Co-production in UK mental health research, lessons from the field / 2018** | Lambert & Carr | UK | Critical reflection on a case study | Researchers with LE and traditional researchers | Paper is cowritten by academic and LER | Discursive paper using a case study to reflect on co-production | Distinguishes coproduction from PPI. Transformative way of thinking about power, resources, partnerships, risks and outcomes, not an off-the-shelf model of service provision or a single magic solution’ | Inclusive research practices | Emancipatory research values provide frameowrk |  |  | Relevant outcomes and processes that value the needs of LE | Systemic barriers, power asymmetries biomedical frameworks. Ethical clearance in being driven by needs of LE and concepts of vulnerability, | All phases | Need for a paradigm shift in services and understanding role of people with LE. Structures are not set up to support co-design so expect resistance. Low expectations. |
| **Co-producing rapid research: Strengths and challenges from a lived experience perspective / 2023** | Machin et al. | UK | Reflections on experiences of research involvment | LER research during COVID | LE researchers within research team | Rapid co-produced research. Reflections on this research | “[. . . ] an approach in which researchers, practitioners, and the public work together, sharing power and responsibility from the start to the end of the project, including the generation of knowledge” (NIHR Involve, 2019, p. 4) | Policy imperitive to foster shared power and decision‐making | 4Pi Framework | Principles in the 4P process, principles purpose presence power | Being realistic about boundaries and constraints. Build mechanisms for evaluating impact | Develop new ways of working and doing codeign using rapid research and adapting 4P framework. | Time, budget, potential for LER to feel like outsiders of a team | All phases | Understanding emotional labour. People with decision making power need to understand LE research and frameworks for effective involvement. Building relationships, time for communication and reflection, coproduction needs to be evaluated and mechanisms for feedback |
| **Co-designing research with Aboriginal and Torres Strait Islander consumers of mental health services, mental health workers, elders and cultural healers / 2022** | Milroy et al. | Australia |  | yes Aboriginal and Torres Strait Islander consumers | yes Co-designing research with Aboriginal and Torres Strait Islander consumers of mental health services, mental health workers, elders and cultural healers | yes culturally sensitive and appropriate research | Aboriginal Participatory Action Research (APAR) |  |  | Consistent with Aboriginal Participatory Action Research. process of working with Aboriginal and Torres Strait Islander peoples in research is as important as the outcome. Aboriginal and Torres Strait Islander leadership, self- determination, and relationship building with commu-nities are essential. it is important for non- Indigenous research team members to be trained to work in cultur-ally safe ways with Aboriginal and Torres Strait Islander peoples. However, it is also important that the entire re-sponsibility of training non- Indigenous researchers does not rest with Aboriginal and Torres Strait Islander mem-bers of the team |  |  |  | all stages of research | taking the time to build respectful partnerships with communities through ongoing consultation, were appropriate and comprehensive methods of co- designing an interview |
| **ALIVE Philosophy of Practice for the Co-design Living Labs: Togetherness by design / 2023** | Palmer et al. | Australia | Retrospective description of the establishment of a community of co-design (Co-design living labs) | Mental health research. LE members include people with lived-experience of mental ill-health and carers/family and kinship group members. Identified the need to have more First Nations involvement | Lived Experience participants in design labs | Co-Design Living Labs network developed to provide a space for LE memberes to co-create research and translation activities. | In the Co-Design Living Labs program, lived-experience is described as and applied as “community-led lived-experience”. This means that people engage as members of the program with their direct, personal experiences of mental ill-health and service systems or support expertise, but there may also be nuances and important elements of lived experience that are located in identities, community stories, events and happenings that are critical to the framing and shaping of experience.” (p. 3) | Focus on participatory design processes, "togetherness by design" (p. 5). Zeitgeist or spirit of our times driving LE within research. | Steps and elements of codeign within the model and table of components provided. | Overarching value/mindset: Togetherness by design drawn from sociologist Zygmund Mauman. The “architecture” or the physical, social, human, and economic infrastructure needed to sustain co-design explored and supported through shared decision making, collective empowerment and championing justice. | Ensuring people were heard, respectful ways of being together, feeling part of the solution. | Identify appropriate research questions, creating a purposeful space, completion of activities in real-life settings. | Researchers need shift power and working with emotions and sharing feelings can be difficult. | End to end involvement of lived experience and establishing priorities for research according to LE views. | Provided as an exemplar of co-design in MH research |
| **Developing relevant community mental health programmes in North India: Five questions we ask when co-producing knowledge with experts by experience / 2023** | Pillai et al. | India | Reflection on coproduction experiences in India | Describe processes of co-production building on the experiences of EBE (people with lived experience of psychosocial or physical disability), practitioners and researchers working together with a non-profit community mental health programme in North India | The paper was developed by practitioners, academics and people with lived experience of mental distress, critically reflecting on their experiences and the journey of co-production of resources and services within a North Indian community mental health initiative. Focus on SU who have intersectional. disadvantage | Used PAR framework | Provided in introduction and use of PAR | Co-production can increase ownership, selfdetermination, and choice for people typically on the receiving end of healthcare. ⇒ Co-production can improve the quality of services and programmes, so that they are more contextually relevant and acceptable | PAR. Questions form stages of research | Co-productive methods are appreciated for their potential to ensure that services are person-centred, cost-efficient, innovative and equitable. Appropriate epistemological and power concerns were addressed when engaging in co-production practices. |  | In settings with limited mental healthcare resources and diverse populations, co-production can increase ownership, self-determination and choice for people typically on the receiving end of healthcare. In all settings, co-production can also improve the quality of tools and programmes, so that they are more contextually relevant and acceptable for those who use services | Time, historical and layered disadvantage in India. How payment was understood eg handout or not feeling deserving because they were not 'experts' | Range | Acknowledging the expertise of EBE requires both social and material resources. The processes of co-production are as important as the products, and require active, attentive, and re.flexive participation by all. Sense of inclusion, mutual social support, improved skills and knowledge, a sense of belonging and collective strength meaning group members are better able to advocate for their own well-being and address upstream social health determinants. These intersect notably with mechanisms of co-production identified elsewhere, as recognition, dialogue, cooperation, accountability, mobilisation, enactment,  creativity and attainment. |
| **Co-production in mental health research: Reflections from the People Study / 2015** | Pinfold et al, 2015 | UK | Reflection | Mental Health Research | University academics, charity-based researchers and a Lived Experience Advisory Panel | Reflections from study research team made up of people with experience of using mental health services, being carers and being academically trained researchers from a range of health and social science disciplines. Some had experience in several areas. The paper is co-produced to provide collective reflection and recommendations | Similarl to user-controlled and other emancipatory methodologies, co-production makes the democratisation of research a primary objective in order to produce better quality and more relevant studies. Co-production also addresses inequalities in power and control within research projects; this way of working does provide a healthy challenge to traditional research hierarchies. In introduction: Boyle and Harris (2009) provide a working definition: “Co-production means delivering public services in an equal and reciprocal relationship between professionals, people using services, their families and their neighbours. Where activities are co-produced in this way, both services and neighbourhoods become far more effective agents of change” (p. 11). | Emancipatory, address systemic power differentials, support recovery and attempt to address the “relevance gap” in academic research and democratise the process | Used published co-production standards | Building equal relationships: recognising people as assets; promoting reciprocity; valuing work differently; building social networks | Communication, clear task boundaries, flexibility, equal remuneration, collective writing | Theoretical benefits and also noted benefits for SU in greater acceptance. Both personal and professional benefits | Time and budget constraints. Ouset was imperfect and did not include SU in determining roles and resources. This impact the rest of the study | all stages | Projects need to have a strategy for how to value different contributions and facilitate constructive relationships if discord emerges. Establishing clear project roles, expectations and process for payment are essential in developing genuine collaborative partnerships. Intersection with Recovery n mental health, co-production principles are increasingly being used to develop recovery-oriented services such as peer worker models (Repper, 2013) |
| **Advancing engagement methods for trials: the CORE study relational model of engagement for a stepped wedge cluster randomised controlled trial of experience-based co-design for people living with severe mental illnesses / 2017** | Richard et al. | Australia | Description of the development of a relational model of engagement | Core study - consumers, carers and staff | Experienced-based co-design (EBCD) methodology called Mental Health Experience Co-design (MH ECO) | CORE stepped wedge RCT | Principles of PAR | Engagement models are essential for research and translation | Experienced-based co-design (EBCD) methodology called Mental Health Experience Co-design (MH ECO | Relational model -giving voice collaboration and empowerment to people on the margins. Ethics of engagement |  | Greater uptake and translation of findings of research with more embedded engagement models. | Barriers to recruitment described | Across all phases on research | Importance of considering the relational world of participants and attention to engagement processes. |
| **Raising the Bar: A Qualitative Study of a Co-Produced Model for Promoting research Partnerships in Mental Health / 2023** | River et al. | Australia | Co-evaluation of a co-produced model of research partnership | LE and conventional researchers | 28 LE and conventional researchers | Aim was to raise the bar in research collaborations towards higher participation. Used qualitative interviews of to explore the research process | A higher level of research participation was defined as continuous participation and equitable decision-making power throughout all stages of the research process, including co-planning, co-design, co-conducting, and co-dissemination | Address epistemic injustice and increase the bar to participation | Raise the Bar model. Three phases Phase 1. Research training for people with lived experience · Phase 2: Development of the Raising the Bar model · Phase 3: Implementation and co-evaluation of the Raising the Bar model | Values are provided within the model and used reference to peer support and models of participation and sharing power and knowledge. | Intentional approach with LE researchers involved at every level. Opportunity to challenge and question. Alterness to issues of power | Meet the needs of affected communities | Structural supports needed including funding and security with employment. Allowing enough time to slow down and do emancipatory process. Risks with using emotional labour | All phases. The paper provides a detailed account of how LE reserchers are involved. Also included coevaluation of the model | Importance of training and practice |
| **Co-production Putting principles into practice in mental health contexts / 2018** | Roper, Grey & Cadogan | Australia | This resource seeks to explain what co-production is, how it is important, how it is different to other participatory approaches, and specific considerations for mental health and offers advice on establishing the culture and mindsets. | Mental health and similar contexts in which extreme power differentials exist. | Contains a number of case studies from different areas. | Unclear how resource was developed. | Gives a definition of co-design within broader co-production | The real difference is how co-production deliberately sets out to create a culture that values all expertise and knowledge, particularly the expertise and knowledge of the people that are most affected by the problem and solution. Co-production recognises and seeks to address power differentials within partnerships. Co-production in mental health, therefore privileges consumer perspective, and promotes and develops consumer leadership, which shifts away from an historical positioning of ‘professionals’ as the experts that steer the agenda. | • Prioritise consumers’ interests  • Support and resource consumers to take the lead on projects  • Consumers set meeting agendas and decide what time should be spent on particular topics/activities  • Consumers could create a ‘deed’ of expectations that everyone in the group signs up to  • Ensure the initiative has a consumer majority  • Establish a consumer steering group to provide governance • Toward the start of meetings, acknowledge people that have experienced or are experiencing a loss of power when engaging with the service system, as a reminder of why power is being closely attended to | The most important part of co-production is shifting mindsets and establishing a culture that embraces exploration and learning, and genuinely values consumer knowledge and expertise. To co-produce means exploring and building (together) the philosophical foundations and mindsets from which  other work can then be done.Co-production identifies, validates and utilises service users’ strengths, supports people’s participation and fosters engagement between services and service users. Thus co—production very much fits within a recovery oriented framework | Seeking consumer leadership in initial thinking and priority setting. Consumer are partners from the inset | Transversal engagement models | Power differentials are acknowledged, explored, and addressed. Within groups that involve both consumer and non-consumer expertise, affirmative actions need to be taken to ensure consumer knowledge and expertise is privileged or else the more powerful group members will influence decisions. | In co-production, consumers are partners throughout all of these stages. co-planning, co-design, co-delivery, and co-evaluation | This resource is comprehensive and contains clear, relevant information. Greater reflection on the limitations of rapid research on coproduction is necessary, as true co-production has always been a slow process |
| **Participatory Action Research-Dadirri-Ganma, using Yarning: methodology co-design with Aboriginal community members / 2021** | Sharmil et al. | Australia | Discusses the process of codesign of a Western methodology (participatory action research) in conjunction with the Indigenous methodologies Dadirri and Ganma. | Australian First Nations people impacted by drug and alcohol and MH | Aboriginal community members and Elders, health professionals and consumers, and non-Indigenous service providers in a drug and alcohol and mental health comorbidity project in Adelaide, South Australia | Process of codesign of a Western methodology (participatory action research) in conjunction with the Indigenous methodologies Dadirri and Ganma. Paper is a reflection on process from Phd student | PAR approach (cycles of Look and Listen; Think and Reflect; Collaborate, Consult and Plan; and Take Action) | Equitable, democratic, liberating and life-enhancing, with real potential to develop feasible solutions. | The resulting combined Participatory Action Research (PAR)-Dadirri-Ganma methodology sought to create a bridge across Western and Aboriginal knowledges, understanding and experiencesWhile the wider CAN project had determined that a PAR approach (cycles of Look and Listen; Think and Reflect; Collaborate, Consult and Plan; and Take Action) was required for each phase [15], the approach and details for the Aboriginal arm of the project were still to be determined. As with the wider project, PAR was selected as the preferred collaborative approach but with the addition of knowledge sharing using Yarning, Dadirri and Ganma  the Look and Listen phase, and Dadirri and Ganma particularly in the Collaborate, Consult and Plan phase as well as throughout the entire project (see Table 1; Figs. 1 and 2 for full details of PAR with Yarning, Dadirri and Ganma methodology and methods) because, from the participants’ perspective, it enabled deeper understanding of the research problem in order to find appropriate and responsive action-oriented solutions [16] | Partnership model for ethical Indigenous research that provided a culturally-safe, holistic, ethically-sound Aboriginal research approach with four key features for creating collaborative engagement with Aboriginal people; ‘Respect’, ‘Collaboration’, ‘Active Participation’ and ‘Meeting Needs’; concepts linked closely to the NHMRC ethical guidelines for Aboriginal research | Foundation pillars of this bridge were mentoring of the PhD student by senior Elders, who explained and demonstrated the critical importance of Yarning (consulting) and Indigenous methodologies of Dadirri (deep listening) and Ganma (two-way knowledge sharing), and discussions among all involved about the principles of Western PAR. Could only do the research if accepted by community | Results focused on needs of service users - informed service improvements and system change. | Researchers need to listen deeply to emotionally distressing information and balance individual issues with the collective needs of diverse groups. | Across a number of phases of research | Attention is needed re. how to learn to elicit the deepest knowledge, truest partnerships and most workable solutions to problems experienced by diverse communities world wide. |
| **Exploring patient and public involvement (PPI) and co-production approaches in mental health research: learning from the PARTNERS2 research programme / 2020** | The PARTNERS2 writing collective | UK | Explores approaches, and specific considerations for mental health using co-operative inquiry approach | Psychosis, spcifically looking at research | Consumer, carer and researchers | Research trials | Coproduction. Used PPI- incorporating experiential knowledge and coproduction as importance of power sharing, promoting inclusive research, value and respecting knowledge and relationahip building. Relinquishing power and doing differently | Policy imperitive and align with best proactice in PPI and coproduction | No model but process included in reflections | Compared to principles of PPI. Values and principles of coproduction | Relationship building, valuing different expertise | No explicit discussion. | Using LE identities of 'normal researchers', identity, discluosure and job titles, them and us division, stigma | Co-operative inquiry method described. | Recognise the importance of emotional work, develop safe spaces, challenges in using personal identities in research, acknowledge power sharing within research and university hierarchy, continual relationship building, valuing different forms of expertise, stigma, transparency in decision making and clear communication. Lived experience expertise- what expertise and how is it applied? Avoid strategic essentialism undermining experiential knowledge into a single cohesive category. importance of diversity, eg culture, and dual perspectives- LE and researcher. Research cannot be fast-tracked. Importance of qualitative methods and story sharing |
| **From monologue to dialogue in mental health care research: reflections on a collaborative research process / 2020** | Tomlinson & De Ruysscher | Belgium | Reflection on research process and draw key lessons. | Mental health | An expert by experience and an academic researcher | A LE researcher and academic researcher share reflections on ethnographic study, data was collected through in-depth interviews with visitors, staff members and volunteers of Villa Voortman. | Dialogical approach to research | Ethical and justice in who owns the story and drives the agenda | Concept of bricolage offered us some theoretical and practical guidance during and after our research process | Dialogue as an ethical imperitive | Organisational culture, personal factors | To move from monological research that reproduces epistemic violence to more dialogical ways of knowledge production |  |  | First of all, for co-creation to take place, experiential knowledge needs to be collectively recognised as a fully valued form of knowledge in the wider debate on recovery and recovery-oriented support. Second, co-creation is much more far-reaching than simply involving the perspectives of persons with lived experience in research (e.g. as a data source and executor of research tasks). Instead, conducting research in dialogical ways implies an intensive, non-linear and relational process that takes place between persons with lived experience and academics. A dialogical research process is one of continuous interaction and bricolage together in all phases of the research trajectory of searching for the most relevant research questions, of seeking out adequate research methods, of keeping an unfinalizable dialogue going about emerging insights and ideas |
| **A qualitative evaluation of coproduction of research: 'if you do it properly, you will get turbulence' / 2021** | Worsley et al. | UK | Evaluation of the process of coproducing a mental health–related research proposal | Mental health service users. Applied health research generally | The working group initially comprised four research professionals and 11 service users, carers and members of the public. Only five public advisors sustained involvement throughout the entire process, becoming co-applicants on the research prop. Included service users and members of the public. | Reflections from members of the public and researchers analysed. Aim to evaluate the process of coproducing a mental health– related research proposal suitable for funding through a national health research funding body. Included semi-structured interviews about the process | PPI definition and use of coproduction | Seen as best process and produce more meaningful research | Steps and elements not described in detail. | Discussed importance of transparency, equal power and mutuality | Good working relationships- open and trusting relationships, equality and shared goals. | that reproduces epistemic violence to more dialogical ways of knowledge | Constrained by time and funding guidelines of funding bodies. Conflict - sense of US and Them. Challenges included professionalised language, jargon, lack of knowledge about the research process, challenges for people in participating, especially when people have experienced trauma and structural challenges, retaining knowledge for some people between sessions, and adhering to funding requirements. Lack of knowledge of the process by researchers. | Meetings to develop a research proposal. | Themes from the interviews- valuing LE perspectives, matching ambitions to funded research process, power relationships and trust, challenges and benefits of coproduction. A recommendation for a separate funding stream for coproduction was needed. Starting each session with healing sessions to build trusted relationahop and reduce negative impacts. Invest in process |
